# SIRT6 Widely Regulates Aging, Immunity, and Cancer

**DOI:** 10.3389/fonc.2022.861334

**Published:** 2022-04-06

**Authors:** Yunjia Li, Jing Jin, Yi Wang

**Affiliations:** ^1^ The First Affiliated Hospital of University of Science and Technology of China (USTC), Division of Life Sciences and Medicine, University of Science and Technology of China, Heifei, China; ^2^ Institute of Immunology and the Chinese Academy of Sciences (CAS) Key Laboratory of Innate Immunity and Chronic Disease, School of Basic Medicine and Medical Center, University of Science and Technology of China, Hefei, China

**Keywords:** aging, immunity, inflammation, cancer, SIRT6, epigenetics

## Abstract

SIRT6 is a member of the Sir2-like family in mammals. Recent structural and biochemical studies have characterized SIRT6 as having deacetylation, defatty-acylation, and mono-ADP-ribosylation activities, which determine its important regulatory roles during physiological and pathological processes. This review focuses mainly on the regulatory functions of SIRT6 in aging, cancer, and, especially, immunity. Particular attention is paid to studies illustrating the critical role of SIRT6 in the regulation of immune cells from the viewpoints of immunesenescence, immunometabolism, and tumor immunology. Owing to its role in regulating the function of the immune system, SIRT6 can be considered to be a potential therapeutic target for the treatment of diseases.

## Introduction

SIRT6 is a multifunctional protein with several enzymatic activities. First, SIRT6 is categorized as a class III histone deacetylase with deacetylation activity (1). However, its substrates are not limited to the acetyl groups of histone H3 and H4 lysine residues but can also include other proteins with acetyl groups on their lysine residues (2), allowing SIRT6 to regulate gene expression or protein activity through post-translational modifications (PTMs). Second, SIRT6 has defatty-acylation activity, which enables it to regulate the secretion of proteins including tumor necrosis factor alpha (TNF-α) (3). Finally, SIRT6 is a mono-ADP-ribosylation enzyme; it can activate poly(ADP-ribose) polymerase-1 (PARP-1), thereby promoting repair of DNA damage (4). These three enzymatic characteristics form the foundation of the ability of SIRT6 to regulate various physiological and pathological processes.

SIRT6 is a longevity protein that can inhibit the aging of cells, tissues, organs and the body by promoting DNA damage repair (5–7), maintaining normal chromosome structure (8–10), and regulating energy metabolism (11, 12) and the senescence-associated secretory phenotype (SASP) (13, 14). Immunosenescence, an aspect of aging, is also inhibited by SIRT6 (15). In addition, SIRT6 can regulate the development of inflammation. At present, most evidence reflects the anti-inflammatory effects of SIRT6 *via* inhibition of the production of inflammatory cytokines and promotion of polarization of immune cells to an immunosuppressive phenotype. For example, SIRT6 has been shown to promote the M2 polarization of macrophages (16–19). However, a few studies have demonstrated pro-inflammatory activity of SIRT6, manifested in the promotion of infiltration and survival of inflammatory immune cells and inflammatory cytokine production (3, 14, 20). SIRT6 also regulates the development process of cancer, an aging-related disease. In melanoma, breast, lung, pancreatic, liver, prostate, colon, ovarian, and blood cancers, SIRT6 has contradictory roles as either oncogene or tumor suppresser at different stages of the cancer or in different tumor cell lines. It shows only tumorigenic effects in osteosarcoma (21) and papillary thyroid cancer (22, 23) and only antitumorigenic effects in bladder cancer (24), nasopharyngeal carcinoma (25), and glioma (26–28). Here, we briefly summarize the enzymatic characteristics of SIRT6; describe in detail the roles of these biochemical and molecular characteristics in aging, immunity, and cancer; and explore the role of SIRT6 in epigenetic immunity and tumor immunology.

## Enzymatic Characteristics of SIRT6

SIRT6 is a member of the Sir2-like protein family. Mammalian sirtuins can be divided into four classes according to their core domain sequences. SIRT6 and SIRT7 are class IV sirtuins (29). SIRT6 has three major enzymatic activities: deacetylation, defatty-acylation, and mono-ADP-ribosylation. These form an important basis for its participation in the regulation of physiological and pathological processes in mammals, including aging, immunity, and cancer occurrence and development.

### Deacetylation Activity

As SIRT6 is located in the nucleus and only diffuses into the cytoplasm (30), it is mainly involved in the regulation of acetylation of lysines on proteins in the nucleus. SIRT6 is a NAD^+^-dependent deacetylase (31). The deacetylation activity of SIRT6 is dependent on nucleosomes, as it adopts its active structure through binding to nucleosomes. When histone H3 and H4 are packaged as nucleosomes instead of free histones, SIRT6 binds to substrates and catalyzes deacetylation (32). Lysine acetylations of multiple sites of histones H3 and H4 are substrates of SIRT6. For example, SIRT6 dynamically combines with chromatin to deacetylate acetylated H3K9 (H3K9ac), thereby regulating telomeric chromatin and inhibiting end-to-end chromosomal fusion and premature aging of cells (31). SIRT6 deacetylates H3K18ac on pericentric heterochromatin to prevent mitotic errors and cell senescence (33). SIRT6 deacetylates H3K56ac. In SIRT6-knockdown cells, hyperacetylation of H3K56 at telomeres was found to influence telomeric chromatin structure, leading to telomere dysfunction and genomic instability (34). SIRT6 can be recruited to the proximal promoter region of the Pcsk9 gene by transcription factor forkhead box O3 (FOXO3), deacetylating H3K9ac and H3K56ac, and thereby inhibiting gene expression (35). Knockdown of SIRT6 in mouse oocytes induces hyperacetylation of H4K16, which significantly increases the incidence of aneuploidy and severely impairs kinetochore-microtubule interactions (36). The deacetylation efficiency of SIRT6 on different substrates is different. SIRT6 can effectively remove the acetyl groups from H3K9, H3K18, and H3K27, whereas its deacetylation activity is weak on H3K4ac, H3K14ac, H3K23ac, H3K36ac, H3K56ac, and H3K79ac (37). In addition to histones, the substrates of SIRT6 include acetylated lysine residues of other proteins, including GCN5 K549ac, PKM2 K433ac, Ku70 K542ac, NAMPT K53ac/369ac, XBP1s K257/297ac, SOD2 K68/122ac, and p53 K382ac (2). The catalytic mechanism of SIRT6-mediated enzymatic function has been well reviewed by Fiorentino et al. (38). Similar to many enzymatic reactions, the catalytic activity of SIRT6 is adjustable. SIRT6 shows poor deacetylase activity *in vitro* (32); however, free fatty acids could increase SIRT6 deacetylation activity 35-fold at physiological concentrations (39).

### Defatty-Acylation Activity

In addition to its deacetylation activity, SIRT6 has defatty-acylation activity, which has emerged as a mechanism regulating the secretion of many proteins. Lysine fatty acylation of TNF-α promotes its lysosomal targeting and degradation (40). A large hydrophobic pocket was identified in the crystal structure of SIRT6 that could accommodate long-chain fatty acyl groups. SIRT6 promotes TNF-α secretion by removing the fatty acyl modification on TNF-α K19 and K20 (3). Furthermore, many ribosomal proteins are secreted by the exosomes of SIRT6-knockout MEFs; this was shown to increase the proliferation of NIH 3T3 mouse embryonic fibroblasts (MEFs) (41). In the same cell line, a SIRT6-knockout mutant showed upregulation of R-Ras2 lysine fatty acylation, which facilitated the localization of R-Ras2 to the plasma membrane and promoted its interaction with phosphatidylinositol 3-kinase (PI3K), thereby activating the Akt signaling pathway and increasing cell proliferation (42).

### Mono-ADP-Ribosylation Activity

SIRT6 is a mono-ADP-ribosyltransferase. Mouse SIRT6 (mSIRT6) relies on NAD^+^ for intramolecular single ADP-riboglycosylation and can be recognized by an antibody specific to mono-ADP-ribose (30). Purified recombinant mSIRT6 has been shown to catalyze radiolabel transfer of [32P] NAD. The transfer of NAD^+^ to mSIRT6 occurs *via* an intra-molecular mechanism, indicating that SIRT6 is an auto-ADP-ribosyltransferase (30). SIRT6 mono-ADP-ribosylates PARP1 K521 to enhance DSB repair (4). It can also mono-ADP-ribosylates BAF170 K312 to promote NRF2 target gene transcription (43). Pan et al. solved the structure of the human SIRT6-ADP-ribose complex (44).

## SIRT6 and Aging

SIRT6 is an important protein with anti-aging effects on cells, tissue, organs, and the body. It inhibits aging *via* four main pathways: promotion of DNA damage repair, maintenance of the normal telomere structure of chromosomes, regulation of glucose and NAD^+^ metabolic balance, and regulation of SASP.

### Physiology and Pathology of SIRT6 in Aging

SIRT6 is a longevity protein that delays the aging process and participates in the maintenance of telomere and genome stability. A study at the organism level showed that SIRT6-deficient mice had small body size, with loss of subcutaneous fat, profound lymphopenia, lordokyphosis, and severe metabolic defects 2–3 weeks after birth, and eventually died at approximately 4 weeks of age (5). SIRT6 deficiency leads to hyperacetylation of histones at the imprinting control region of developmental repressor H19, which results in severe prenatal developmental delay and death several hours after birth in SIRT6-deficient monkeys (45). By contrast, transgenic mice overexpressing SIRT6 have a longer life span than wild-type mice (11, 46). A study at the organ and tissue levels also demonstrated the anti-aging activity of SIRT6 and proposed SIRT6 as a potential marker of ovarian aging. Its expression was positively correlated with the number of primordial follicles; both SIRT6 protein expression and ovarian reserves decreased with increasing age (47). SIRT6-deficient mice showed symptoms of myocardial hypertrophy and heart failure, and the expression of SIRT6 in failing human hearts was reduced (48).

Cell-level studies have also shown that SIRT6 inhibits cell senescence. SIRT6 could reduce cardiac hypertrophy and cardiomyocyte senescence (49). The addition of SIRT6 increased the resistance of elderly human dermal fibroblasts to classic Yamanaka factor-induced reprogramming (50). SIRT6-deficient human mesenchymal stem cells exhibited accelerated functional decline, which was mainly characterized by redox metabolism disorders and increased sensitivity to oxidative stress and was different from typical cell senescence (51). By contrast, overexpression of SIRT6 inhibited the replicative senescence of chondrocytes (52).

### Mechanism of SIRT6 in Anti-Aging

Cell and molecular biology can explain the anti-aging effect of SIRT6. Firstly, SIRT6 is a nucleolar chromatin-related protein involved in various DNA damage repair processes. SIRT6 is related to base excision repair (BER), which has been shown to promote resistance to DNA damage in mouse cells, suppress genomic instability, and promote normal DNA recombination (5). Mechanistically, SIRT6 activates PARP1 through mono-ADP-ribosylation activity and interacts with two BER enzymes (hMYH and hAPE1) to promote BER (6, 53). SIRT6 was recruited to ultraviolet-induced DNA damage sites and deacetylated damaged DNA binding protein 2 (DDB2) at K35 and K77, promoting segregation of DDB2 from chromatin and thereby facilitating nucleotide excision repair (NER) (7). In mammalian cells, oxidative stress activates the protein kinase c-Jun N-terminal kinase to phosphorylate SIRT6 at serine 10; SIRT6 is then recruited to DNA double-strand break (DSB) sites. SIRT6 mono-ADP-ribosylates the K521 lysine of PARP1; this activates PARP1 and enhances DSB repair under oxidative stress (4, 54). SIRT6 recruits chromatin recombinant SNF2H to the DNA cleavage site and deacetylates histone H3K56ac, preventing genomic instability through chromatin remodeling and facilitating the repair of damaged sites (55).

Secondly, SIRT6 can maintain the normal chromosome structure. The stability of chromosomes and telomeres is particularly important for cell anti-aging (56). SIRT6-deficient cells exhibit abnormal telomere structures, including increased chromosomal fragmentation, detached centromeres, and chromosomal gaps, similar to cell defects observed in Werner syndrome (a premature aging disorder) (5, 31). The deacetylation activity of SIRT6 plays an important part in maintaining genome stability. SIRT6 deacetylates H3K9ac, stabilizes the binding of WRN to telomeric chromatin to resist replication-related telomere defects, and prevents end-to-end chromosomal fusion and premature senescence of cells (31). SIRT6 interacts with the nuclear factor-κB (NF-κB) RELA subunit and deacetylates H3K9ac at the promoter of the NF-κB target gene to suppress cell senescence (8). SIRT6 promotes H3K18ac deacetylation, silences pericentric heterochromatin at centromeres, and prevents aberrant accumulation of pericentric transcripts (33). SIRT6 is necessary for maintaining the silencing of the telomere position effect in human cells and plays a key part in maintaining the structure of silent telomeric chromatin (9). As a powerful repressor of retrotransposon L1, SIRT6 mono-ADP-ribosylates KRAB-associated protein 1 (KAP1) and promotes the interaction between KAPI and heterochromatin protein 1 alpha (HP1α), thereby contributing to the packaging of L1 gene elements into heterochromatin to reduce their expression (10).

Thirdly, in addition to maintaining genomic stability as mentioned above, SIRT6 slows the process of aging by regulating glucose homeostasis and the NAD^+^ metabolic balance (11, 12). Overexpression of SIRT6 is conducive to a “young state” of blood glucose and gluconeogenesis in aged mice. SIRT6 promotes hepatic gluconeogenesis by increasing lipolysis, and increases the levels of precursors of the gluconeogenesis and tricarboxylic acid (TCA) cycles, thereby maintaining the young state of these two cycles (11). In addition, SIRT6 is helps to maintain NAD^+^ levels by increasing the expression of *de novo* NAD^+^ synthesis genes (11). However, SIRT6 is a NAD^+^-dependent enzyme and also consumes NAD^+^ during the processes of deacetylation (38), defatty-acylation (42), and mono-ADP-ribosylation (30). NAD^+^ can delay aging by inhibiting P53 activity. Disruption of the NAD^+^/NADH ratio in cell solute could promote aging through mitochondrial dysfunction (12). The above conclusions are described in detail in Roichman's research (11) and Wiley’s review (12).

Finally, some studies have shown that SIRT6 affects the SASP to prevent aging. Aging is closely related to the immune system, and immunosenescence is a part of aging. Immune cell components, functions, and intercellular interactions in the innate and adaptive immune systems tend to develop immunotolerance in the process of immunosenescence (57, 58). SIRT6 levels in the articular chondrocytes of osteoarthritis patients are significantly reduced; therefore, overexpression of SIRT6 could reduce the senescence of chondrocytes and prevent the development of osteoarthritis (52). Studies have shown that SIRT6 is directly involved in the regulation of immunosenescence. Dendritic cell (DC) dysfunction is at the core of various common chronic diseases and contributes to the reduction in immunocompetence that occurs during aging. SIRT6-knockout (SIRT6KO) mice showed a lower frequency of bone marrow conventional DC (cDC) precursors and lower numbers of bone marrow-derived cDCs. SIRT6KO mouse cDCs expressed low levels of MHCII, chemokine receptor CCR7, and costimulatory molecules and had lower immunostimulatory activity than wild-type cDCs. The ability of SIRT6KO cDCs to produce IL-12 was generally reduced. SIRT6 deficiency prevented the maturation of BMDCs (bone marrow-derived DCs) generated *in vitro* in a partial TNF-α-dependent manner. SIRT6 helps BMDCs respond to Toll-like receptor (TLR) ligands. The proliferation of allogeneic lymphocytes in a mixed leukocyte reaction (MLR) stimulated by cells cultured in the presence of SIRT6 inhibitors was also significantly reduced. Therefore, SIRT6 plays a crucial part in the differentiation and function of cDCs, and loss of function of SIRT6 may promote immunosenescence (15). Inflamm-aging (a long-term low levels of inflammatory mediators in aging individuals) (57) will follow. This is often accompanied by the production of a SASP (senescent cells secrete pro-inflammatory cytokines, chemokines, and proteases) (13). SIRT6 increases TNF secretion in BMDCs and THP-1 through post-transcriptional steps (3, 14). Overexpression of SIRT6 proved sufficient to delay the replicative senescence of diploid fibroblast WI38 by attenuating NF-κB signaling. Knockdown of SIRT6 leads to accelerated cell senescence and overactivation of NF-κB (59). Therefore, SIRT6 affects the NK-κB pathway, which regulates cytokine production (discussed in more detail later), and then regulates SASP. In short, SIRT6 may also participate in inflamm-aging *via* affects on the SASP through regulating the synthesis and release of inflammatory factors.

## SIRT6 and Immunity

SITR6 regulates inflammatory development, and plays a complex role. It inhibits inflammatory by promoting M2 macrophage polarization, decreasing number of lymphocytes, inhibiting T cell differentiation, and inhibiting innate immunity response. However it also promotes inflammatory by promoting neutrophils and dendritic cells migration, and promoting TNF-α secretion. Besides, SIRT6 could regulate immunometabolism. All of these will be discussed in detail below.

### SIRT6 Regulates Inflammatory Development

Some studies have shown that SIRT6 can inhibit inflammation. In myeloid cells, most studies have focused on the role of SIRT6 in regulating macrophage polarization. Sirt6^f/f^:Fabp4-Cre mice (with SIRT6 deficiency in preadipocytes and mature adipocytes) exhibited increased expression of inflammatory genes including *F4/80, Tnfα, Mcp-1*, and *Il6* in both brown and white adipose tissues (16). Adipocyte-specific S6KO mice showed increased infiltration of macrophages in epididymal white adipose tissue, many M1 macrophage genes (*Il1b*, *Ccl2*, *Tnfα*, *Il6*, *Nos2*, and *Ccr2*) were upregulated at the mRNA level, and inflammation was increased. However, the mRNA levels of M2 macrophage genes (*Mrc1*, *Mgl1*, *Arg1*, and *Il10*) were downregulated (17). In the same mouse model, macrophages infiltrated into adipose tissue. Moreover, the ratio of M1/M2 macrophages was significantly increased, and the mRNA expression of inflammatory genes including *CD11b^+^*, *Cxcl2*, *CD68*, *Tnfα*, *Mcp-1*, and *Il6* was also increased when mice were fed a high-fat diet (18). By contrast, SIRT6 regulated the expression of IL-4 by adipocytes through an autocrine route, thereby promoting the polarization of macrophages to the M2 type and reducing inflammation (17). In a full-thickness excisional lesion model of dorsal skin, the infiltration of M1 macrophages in myeloid-cell-specific S6KO (mS6KO) mice increased, whereas numbers of M2 macrophages decreased. Therefore, inflammation at the wound site increased, and wound healing was impaired. By contrast, under the condition of M2 polarization stimulated by IL-4, transducing mS6KO bone marrow macrophages (BMMs) with adenovirus expressing Sirt6 promoted the polarization of M2 macrophages by protecting the PI3K-Akt pathway (19). Clinical analysis has shown that levels of SIRT6 in particular chondrocytes of osteoarthritis patients are significantly reduced. Expression levels of SIRT6 in peripheral blood mononuclear cells, monocytes, and macrophages are lower in patients with rheumatoid arthritis compared with those with osteoarthritis. The activity of SIRT6 is negatively correlated with the severity of the disease. Overexpression of SIRT6 can reduce the inflammatory response by reducing the expression of NF-κB-dependent genes, thereby preventing the development of arthritis (52, 60). The arthritis of mS6KO mice is more serious than that of wild-type mice. Lack of SIRT6 leads to upregulation of acetylated-FoxO1 protein levels and CCR3 expression in macrophages, and the migration of macrophages to synoviocyte-derived chemoattractants is enhanced so that more macrophages gather in the synovium (60). In collagen-induced arthritis (CIA), overexpression of SIRT6 inhibits the differentiation of osteoclasts in BMDCs induced by macrophage colony-stimulating factor. The severity of arthritis is reduced, and levels of local and systemic pro-inflammatory cytokines are also downregulated (61). In neuro-inflammation, SIRT6 activation inhibits lipopolysaccharide (LPS)-stimulated inflammatory responses of RAW264.7 macrophages and primary mouse microglia (62). *In vitro* experiments have also confirmed the anti-inflammatory effect of SIRT6. SIRT6-deficient macrophages promote the activation of NF-κB and the production of IL-6, which results in signal transducer and activator of transcription 3 (STAT3) activation and a positive feedback loop for NF-κB stimulation, and finally accelerates the polarization of pro-inflammatory M1 macrophages (63). By contrast, icariin (ICA) upregulates the expression and increases the activity of SIRT6. ICA treatment inhibits the NF-κB inflammatory signaling pathway and reduces mRNA levels of the NF-κB downstream target genes: *Tnfα*, *Il2*, *ICAM-1*, and *Il6*, thereby inhibiting inflammatory development (64). BMMs of Sirt6-null mice show high expression of pro-inflammatory genes encoding MCP-1, TNF-α, and IL-6 and hypersensitivity to LPS stimulation due to the hyperacetylation of H3K9 and the increase in c-JUN occupancy in the promoters of these genes (65). By contrast, macrophages overexpressing SIRT6 are transformed into the M2 phenotype, which can avoid the damage induced by high glucose levels (66). S6KO mice have abnormal immune systems and metabolism. There are inextricable links between immune cells and immune cells, and between metabolism and immune cells. It is thus difficult to accurately study a particular type of immune cell under these conditions of multifactorial changes.

Studies have also shown that SIRT6 inhibits inflammation *via* effects on lymphocyte differentiation and function. In S6KO mice, lordokyphosis, colitis caused by erosion of the superficial colonic epithelium, acute loss of subcutaneous fat, and severe lymphopenia relevant to increased lymphocyte apoptosis were observed. Flow cytometry analysis showed that in the thymus, the number of CD4^+^-CD8 ^+^ double-positive cells decreased 50-fold, and in bone marrow, the number of splenic lymphocytes and progenitor B cells decreased 10-fold (5). In CIA rats, a low percentage of regulatory T cells (Tregs) was observed following treatment with C3G (an inhibitor of CD38) and the Sirt6 inhibitor OSS_128167 (67). SIRT6 interacts with and deacetylates GATA3, inhibits the Th2 immune response, and reduces the expression of IL-4, IL-5, and IL-13, thereby weakening airway allergic inflammation induced by ovalbumin or house dust mites (68). S6KO mice developed chronic liver inflammation at approximately 2 months old, and the absence of SIRT6 in T cells was sufficient to induce liver fibrosis and inflammation (65).

Furthermore, SIRT6 negatively regulates the innate immune response during dengue virus (DENV) infection. SIRT6 silencing enhances the production of pro-inflammatory cytokines and chemokines. Overexpression of SIRT6 inhibits NF-κB activation mediated by RIG-I-like receptor and TLR3. The sirtuin core domain of SIRT6 is important for inhibiting NF-κBp65 function. SIRT6 interacts with the p65 DNA-binding domain and competes with p65 to bind the IL-6 promoter and reduce the expression of IL-6 during DENV infection (69).

However, some studies have confirmed that SIRT6 promotes inflammatory development and has pro-inflammatory potential. Human SIRT6 promotes TNF-α secretion by removing the fatty acyl modifications on TNF-α K19 and K20 (3). After CpG stimulation of BMDCs from SIRT6-deficient mice, the amounts of TNF-α synthesized by the cells decreased, which confirmed the pro-inflammatory effect of SIRT6 (14). In autoimmune diseases, SIRT6 inhibitors effectively delay the onset of experimental autoimmune encephalomyelitis (EAE) *via* the following mechanism: inhibition of SIRT6 reduces the expression of CD40 on lymph node DCs, decreases encephalitogenic T cell infiltration, and decreases the ability of CXCR4^+^ DCs to migrate into the lymph nodes of EAE mice. Levels of IFN-γ, TNF-α, and IL-12 were also decreased, but the expression of IL-10 was increased with an anti-inflammatory effect (20). SIRT6 also increased the levels of cAMP/Ca^2+^-dependent transcription factors and nuclear factor of activated T cells through its deacetylation activity, thereby enhancing the expression of TNF- α and chemokine IL-8 (70).

### SIRT6 Regulates Immunometabolism 

Many studies have confirmed that SIRT6 is involved in cell metabolism regulation (71). Metabolism is an important factor affecting the development and function of immune cells (72, 73). For example, Shun et al. reported that hypoxia may stimulate cell glycolysis and autophagy, and that autophagy promotes the formation of DNA-containing immune complexes and trafficking of TLR9 to the signaling compartment, leading to hyper-responses of immune cells, which are related to the formation of nasal polyps (74–76). The expression of SIRT6 is inhibited in a chronic inflammatory state. If SIRT6 expression is increased, autophagy can be inhibited by inhibiting anaerobic glycolysis, which is conducive to disease treatment (77).Cyclosporine A inhibits neutrophil migration and apoptosis by inhibiting SIRT6, promoting the upregulation of HIF-1α expression and enhancing glycolysis and the TCA cycle, which is conducive to the remission of acute severe ulcerative colitis (78). During the transformation from early inflammation to late inflammation, SIRT6 deacetylates H3K9ac and H3K56ac on several glycolysis gene promoters, inhibits HIF-1α transcriptional activity, and reduces glycolysis activity to promote M2 polarization of macrophages (79).

SIRT6 might regulate T cell differentiation by metabolism. SIRT6 deacetylates and activates FoxO1 to regulate lipid metabolism in brown adipocytes (80). The absence of FoxO1 seriously inhibits the development of Foxp3^+^ Tregs (81). SIRT6 inhibits HIF-1α activation to inhibit glycolysis, and HIF-1α activates RORγt to enhance Th17 cell polarization (82). HIF-1α promotes IL-9 expression to induce Th9 cell polarization (83). It is well known that after challenge by pathogens, some T cells will continue to exist as longevity memory T cells, which maintain self-renewal capacity, allowing them to proliferate many times over a long duration to prevent rechallenge by the same pathogen. They are long-lived immune cells (84, 85). Unlike naive CD8^+^ T cells, the percentages of effector memory CD8^+^ T cells and central memory CD8^+^ T cells increased with age (86). Subsequently, researchers found that the central memory cells of older individuals shifted toward a chromatin-opening pattern and determined that the gene regulation driven by NRF1 and BATF was a potential target for delaying CD8^+^ T cell aging (87). Other studies have shown that NRF-1 itself is regulated by PTMs (acetylation, methylation, and phosphorylation) that enhance binding to its target genes (88). The above results suggest that SIRT6 may be involved in the accumulation of memory CD8^+^ T cells in the aging population because of its capacity to deacetylate.

Although few studies have shown SIRT6 to affect immune cell function through directly regulating immunometabolism, SIRT6 has been found to regulate immune cell activity *via* effects on PI3K/Akt, NF-κB, and HIF-1α as discussed above. The above three signaling pathways could be involved in regulation of the TCA cycle, glycolysis, pentose phosphate pathway, and Warburg effect, thereby affecting the energy metabolism of immune cells in the resting state and activated state (72, 89–94). SIRT6 also regulates intracellular levels of NAD^+^, which is an electron acceptor with a key role in cell metabolism. Therefore, SIRT6 is likely to regulate immune cell proliferation, growth, and function by affecting immunometabolism. Pillai’s review discusses this point in detail and proposes that SIRT6 participates in short-term regulation of immune cells through PTMs and long-term regulation through transcriptional regulation of metabolism-related genes (89). Therefore, how SIRT6 regulates immunometabolism and how this regulation affects cell function will be an interesting research direction.

## SIRT6 and Cancer

On the one hand, frequent DNA damage and mutation will lead to canceration (95, 96), which provides important support for the development of tumors; on the other hand, chronic inflammation caused by aging will drive tumor initiation, growth, progression, and metastasis (97). Moreover, immunosenescence is conducive to escape of cancer cells from immune system attacks and their eventual development into cancer. As mentioned earlier, SIRT6 plays a positive role in maintaining genomic stability and preventing aging. So SIRT6 is involved in cancer regulation is obvious.In addition, large amounts of data indicate that SIRT6 is directly involved in the occurrence and development of cancer.

### SIRT6 Promotes/Inhibits Cancer Development

In certain cancers, SIRT6 promotes cancer development. In osteosarcoma, inhibition of SIRT6 enhances the antitumor effect of doxorubicin by inhibiting the DNA damage repair pathway (21). In papillary thyroid cancer (PTC), SIRT6 increases generation of reactive oxygen species to promote the Warburg effect in PTC cells, and high levels of SIRT6 reduce expression of E-cadherin, thereby promoting the invasion and migration of PTC cells and promoting cancer development (22, 23).

However, in other cancers, SIRT6 exhibits tumor suppressor activity. When bladder cancer develops from T2 to T4, the expression of SIRT6 is significantly decreased. Low SIRT6 expression increases the acetylation of H3K9 and levels of Glut1 and PDK1, enhances glycolysis, and increases the proliferation ability of tumor cells (24). In nasopharyngeal carcinoma, SIRT6 overexpression reduces levels of anti-apoptotic protein Bcl-2 but increases levels of cleaved caspase-3 and pro-apoptotic protein Bax. High levels of SIRT6 inhibit NF-κB signaling and promote apoptosis of nasopharyngeal carcinoma cells (25). SIRT6 is downregulated in human glioma tissues and deacetylates H3K9ac on the promoter of PCBP2 to downregulate PCBP2 expression and inhibit glioma cell growth (26). Elevated SIRT6 expression leads to tumor cell apoptosis by upregulating the expression of Bax and cleaved caspase-8, and downregulating Bcl-2, and inhibiting the Janus kinase 2 (JAK2)-STAT3 pathway (27). FOXO3a transcriptionally activates SIRT6 to inhibit the Warburg effect in glioblastoma cells, thereby inhibiting the development of glioblastoma (28).

### The Dual Role of SIRT6 in Cancer Development

In melanoma, CRISPR/Cas9 or lentivirus short hairpin RNA-mediated knockout or knockdown of the SIRT6 gene in A375 melanoma cells, leding to significantly reduced growth, vitality, and clonogenic survival rates of cancer cells, induced cell cycle arrest in G1 phase, and increased senescence-associated β-galactosidase staining (98, 99), reflecting the oncogenic activity of SIRT6. However, in BRAF^V600E^ melanoma cells, SIRT6 haploinsufficiency induced resistance of melanoma cells to mitogen-activated protein kinase (MAPK) inhibitors by activating IGF signaling (100), suggesting an anti-tumor effect of SIRT6. The expression of SIRT6 is decreased in primary melanoma compared with melanocytic nevus. An increase in SIRT6 induces inhibition of cell proliferation, cell cycle arrest, and apoptosis. However, in the metastatic stage of melanoma, the expression of SIRT6 increases (possibly induced by FOXO3a) and promotes the development of melanoma in an autophagy-dependent manner by inhibiting IGF-AKT signaling (101, 102).

In breast cancer, SIRT6 can enhance the expression and activity of pyruvate dehydrogenase (PDH), thereby enhancing oxidative phosphorylation in breast cancer cells and promoting the occurrence of breast tumors in mice (103). High nuclear levels of SIRT6 promot cancer development and is significantly associated with poor overall survival (104). Low levels of SIRT6 increase acetylated FOXO3, thereby inhibiting tumor development (105). However, another study showed that ectopic expression of SIRT6 reduced pAkt, hexokinase-2, and PDH kinase-1 protein levels, thereby inhibiting metabolic pathways in breast cancer (106).

In lung cancer, SIRT6 is overexpressed in non-small-cell lung cancer (NSCLC) cell lines (107–109). SIRT6 increases extracellular signal-regulated kinase (p-ERK) 1/2 phosphorylation and activates matrix metalloproteinase 9 (MMP9) to facilitate tumor cell migration and invasion (109). Silencing of SIRT6 impaired the proliferation and differentiation of NSCLC cell lines, arresting cells in the S and G0/G1 phases (107). miR-34 inhibited the proliferation of A549 cells by inhibiting SIRT6 expression (108). A lack of SIRT6 leads to upregulation of Kruppel-like factor 4 (KLF4) in NSCLC cells to reduce their invasiveness (110). However, studies have shown that patients with low nuclear expression of SIRT6 have cancer that is more aggressive and shorter survival (111). SIRT6 inhibits cell proliferation by inhibiting the expression of Twist1 in NSCLC (112). In the A549 lung cancer cell line, α-hederin was shown to inhibit c-Myc and HIF-1α by increasing the expression of SIRT6 to inhibit glycolysis and further inhibit the proliferation of A549 cells (113).

In pancreatic cancer, the SIRT6 inhibitor quinazolinedione synergistically kills pancreatic cancer cells with gemcitabine (114). SIRT6 enhances Ca^2+^ responses, which promotes the migration ability of pancreatic cancer cells (70). However, another study found that SIRT6 was an important tumor suppressor in pancreatic ductal adenocarcinoma. The absence of SIRT6 leads to hyperacetylation of the Lin28b promoter, Myc recruitment, and significant induction of Lin28b and its downstream let-7 target genes *IGF2BP1*, *IGF2BP3*, and *HMGA2*, thereby promoting the development of cancer (115).

In liver cancer, upregulation of SIRT6 is very common in liver cancer tissues and is highly correlated with poor overall survival rate, disease-free survival, hepatocellular carcinoma (HCC) cell migration, tumor size, tumor grade, and vascular invasion (116, 117). The suppression of SIRT6 in various liver cancer cell lines can inhibit cell growth and induce apoptosis *in vitro. In vivo* experiments also confirmed that the suppression of SIRT6 inhibits tumor growth (117). SIRT6 promotes the migration, invasion, and epithelial–mesenchymal transition (EMT) of HCC cells. Mechanistically, SIRT6 overexpression induces E-cadherin degradation to improve cancer invasion and migration ability. SIRT6 deacetylates the promoter of Bax (the main determinant of apoptosis of cancer cells) at H3K9 and suppresses its promoter activity to prevent cancer cell apoptosis (117). SIRT6 reduces the acetylation of AKT, resulting in increased phosphorylation of AKT and promoting its activity. Activated AKT promotes phosphorylation of anti-apoptotic protein X-linked inhibitor of apoptosis protein to prevent cancer cell apoptosis (118). SIRT6 silencing inhibits the growth of HCC cell lines by inducing p53/p21- and p16/Rb-independent cell senescence (119). However, some studies have found that SIRT6 inhibits the development of liver cancer. The level of SIRT6 decreases with increasing liver cancer grade, and increasing the level of SIRT6 at the initiation stage could significantly impair the development of cancer (120, 121). Mechanistically, the decrease in SIRT6 levels increases the acetylation level of the lysine residue at position 433 of nuclear pyruvate kinase M2 (PKM2) and promotes the oncogenic functions of PKM2, which is conducive to cell proliferation, migration, and invasion (120). Increasing SIRT6 levels represses survivin and inhibits cancer progression by reducing histone H3K9ac and NF-κB activation (121).

In prostate cancer, SIRT6 is overexpressed in prostate tumors. Knockdown of SIRT6 in prostate cancer cells results in cell cycle arrest at sub-G1 phase, increased apoptosis, increased DNA damage, and decreased BCL2 expression, thereby reducing cancer cell viability and enhancing chemotherapeutic sensitivity (122). The absence of SIRT6 significantly inhibits the activation of prostate cancer-related signaling pathways such as the Notch pathway, thereby inhibiting the proliferation and metastasis of prostate cancer cell lines (123). However, studies have shown that E2F promotes tumor growth by suppressing SIRT6 transcription to enhance glycolysis (124).

In colon cancer, SIRT6 deacetylates H3K9ac to promote the EMT process by reading snail and inhibiting TET1 transcription, further promoting tumorigenesis. Knockdown of SIRT6 in HCT116 cells leads to reduced colony formation (125). However, studies have shown that the expression of SIRT6 protein in colon cancer tissues is downregulated, and patients with higher SIRT6 expression show better prognosis (126). Upregulation of SIRT6 promoted the expression of PIP2 and PTEN and improved the stability of PTEN. The apoptosis levels of SW620 colon cancer cells overexpressing SIRT6 increased, and their proliferation ability was weakened (127). USP10 protects SIRT6 from proteasome-mediated degradation. SIRT6 inhibits c-Myc transcriptional activity, thereby inhibiting cell cycle progression, cancer cell growth, and tumor initiation in the colon cancer cell line HCT116 (128).

In ovarian cancer, SIRT6 knockdown in OVCAR3 and OVCAR5 ovarian cancer cells significantly inhibited cell migration and invasion (129). However, the expression of SIRT6 in human ovarian cancer tissues was significantly decreased, and the expression of Notch3 was increased, which further promoted the development of cancer (130).

In addition to its role in solid tumors, SIRT6 regulates blood cancer in a similarly complex manner. SIRT6 is overexpressed in CD34^+^ hematopoietic progenitors and multiple myeloma in patients with acute myeloid leukemia, and high SIRT6 levels are associated with poor prognosis (131, 132). SIRT6 deacetylates DNA-Pkc and CtIP and inactivates ERK2/p90RSK signaling to increase DNA repair, conferring DNA damage resistance (132). In diffuse large B-cell lymphoma cells, knockdown of SIRT6 increased sensitivity to chemotherapy, apoptosis rates, dysfunctional cell proliferation, and cell cycle arrest between the G2 and M phases, reflecting the tumor-promoting activity of SIRT6 (133). However, some studies have shown that SIRT6 deacetylates H3K9ac at the promoter of transcription factor ELK1 and ERK signal-related genes, thereby downregulating the signal transduction of the MAPK pathway and decreasing proliferation (132).

In summary, SIRT6 has shown contradictory results, promoting or suppressing cancer among different cancers, and even at different stages of development or different cell lines of the same cancer (**Table 1**). If SIRT6 is to be used to regulate the development of cancer *via* effects on the metabolism, proliferation, and apoptosis of cancer cells, it will be necessary to conduct a very comprehensive study of its role in the occurrence and development of various cancers. Effective activators and inhibitors of SIRT6 are also needed to suit the remedy to the case. As SIRT6 also has important roles in aging and immune regulation, how to reduce drug side effects will be another urgent problem to be solved in the future. At present, few studies have shown whether SIRT6 could enhance the anti-tumor ability by regulating the activity of immune cells; this may become a new research direction in the future.

**Table 1:** Regulatory mechanisms of SIRT6 in various cancers.

Cancer type	Function	Mechanisms
**Osteosarcoma**	**Oncogene** **Oncogene**	DNA damage repair
**Papillary thyroid cancer**		Promotes the Warburg effect Decreases E-cadherin expression
**Bladder cancer**	**Suppressor**	Decreases GLUT1 and PDK1 to inhibit glycolysis
**Nasopharyngeal carcinoma**	**Suppressor**	Decreases Bcl-2 levels Increases Bax and cleaved caspase-3 levels Inhibits NF-κB signaling
**Glioma**	**Suppressor**	Suppresses expression of PCBP2 Inhibits JAK2/STAT3 signaling
		Inhibits the Warburg effect
**Skin cancer**	**Oncogene**	Promotes COX-2 expression Protects cell cycle progression
	**Suppressor**	Inhibits IGF-AKT signaling
**Breast cancer**	**Oncogene**	Enhances oxidative phosphorylation
		Suppresses FoxO3 activity
	**Suppressor**	Suppresses glucose metabolism
**Lung cancer**	**Oncogene**	Increases p-ERK1/2 and activates MMP9 Protects cell cycle progression Suppresses KLF4 expression
	**Suppressor**	Inhibits Twist1 expression Inhibits glycolysis
**Pancreatic Cancer**	**Oncogene**	Enhances Ca^2+^ responses
	**Suppressor**	Inhibits Lin28b and downstream let-7 target genes
**Hepatocellular Carcinoma**	**Oncogene**	Promotes EMT by stimulating autophagic degradation of E-cadherin
		Suppresses Bax expression
		Increases phosphorylation and activity of AKT
		Prevents DNA damage and cell senescence
	**Suppressor**	Suppresses nuclear localization of PKM2
		Activates NF-κB and inhibits survivin
**Prostate cancer**	**Oncogene**	Protects cell cycle progression
		Promotes Bcl2 expression Protects Notch signaling pathway
	**Suppressor**	Inhibits glycolysis
**colon cancer**	**Oncogene**	Reads Snail and suppresses TET1 transcription to promote EMT
	**Suppressor**	Promotes the expression of PIP2 and PTEN
		Inhibits c-Myc transcription
**Leukemia**	**Oncogene**	Protects cell cycle progression Repairs DNA damage through DNA-PKc/CtIP and ERK2/p90RSK signaling
	**Suppressor**	Inhibits MAPK signaling pathway
**Ovarian cancer**	**Oncogene**	Promotes EMT
	**Suppressor**	Downregulates Notch 3 expression

## Conclusion and Future Perspectives

SIRT6 has a range of PTM capabilities including deacetylation, defatty-acylation, and mono-ADP-ribosylation activities. This multifunctional PTM protein is widely involved in aging, immunity, and cancer regulation. The substrates of SIRT6 during aging, immunity, and cancer regulation are summarized in **Table 2**.

**Table 2:** Substrates and enzymatic activity of SIRT6 during aging, immunity, and cancer regulation.

	Substrates	Enzymatic activity
**Aging**	PARP1 K521	Mono-ADP-ribosylation
	DDB2 K35 and DDB2 K77	Deacetylation
	H3K56ac at DNA damage sites	Deacetylation
	H3K9ac in the promotor of NF-κB	Deacetylation
	H3K18ac in pericentric chromatin	Deacetylation
	KAP1	Mono-ADP-ribosylation
**Immunity**	H3K9ac in the promoter of NF-κB	Deacetylation
	Enhancer of Zeste homolog 2	Deacetylation
	FOXO1	Deacetylation
	Pyruvate kinase muscle isozyme	Deacetylation
	H3K9ac in the promoters of *Il-6* and *Mcp-1*	Deacetylation
	GATA3	Deacetylation
	H3K9ac in promoters of HIF-1α target genes	Deacetylation
	FOXO1	Deacetylation
	TNF-α K19 and TNF-α K20	Defatty-acylation
**Cancer**	H3K9ac in the promoter of *PCBP2* gene	Deacetylation
	H3K56ac at the IGFBP2 locus	Deacetylation
	H3K9ac in the promoters of a cluster of glycolysis-associated genes	Deacetylation
	Snail	Deacetylation
	H3K9ac, H3K56ac	Deacetylation
	H3K9ac in the promoter of *Bax*	Deacetylation
	Beclin-1	Deacetylation
	AKT	Deacetylation
	PKM2	Deacetylation
	H3K9ac in the promoter of *survivin*	Deacetylation
	H3K9ac in the promoter of *Erk2*	Deacetylation
	H3K56ac at DNA damage sites	Deacetylation
	R-Ras2	Defatty-acylation

**Figure 1:**
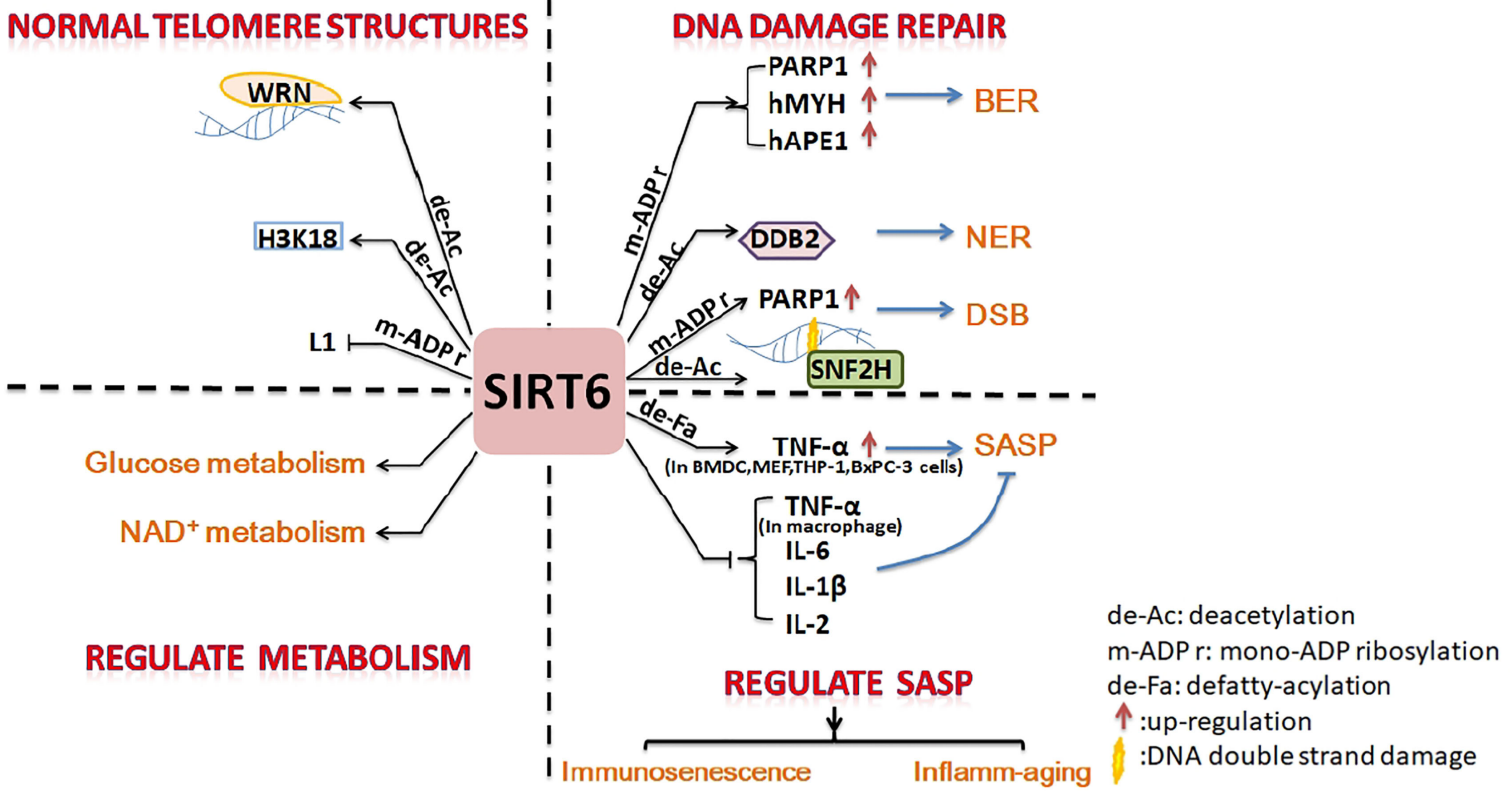
Summary of inhibition of cell senescence by SIRT6. SIRT6 promotes DNA damage repair, maintains the normal structure of telomere chromatin, regulates energy metabolism, and inhibits SASP to prevent cell senescence.

**Figure 2:**
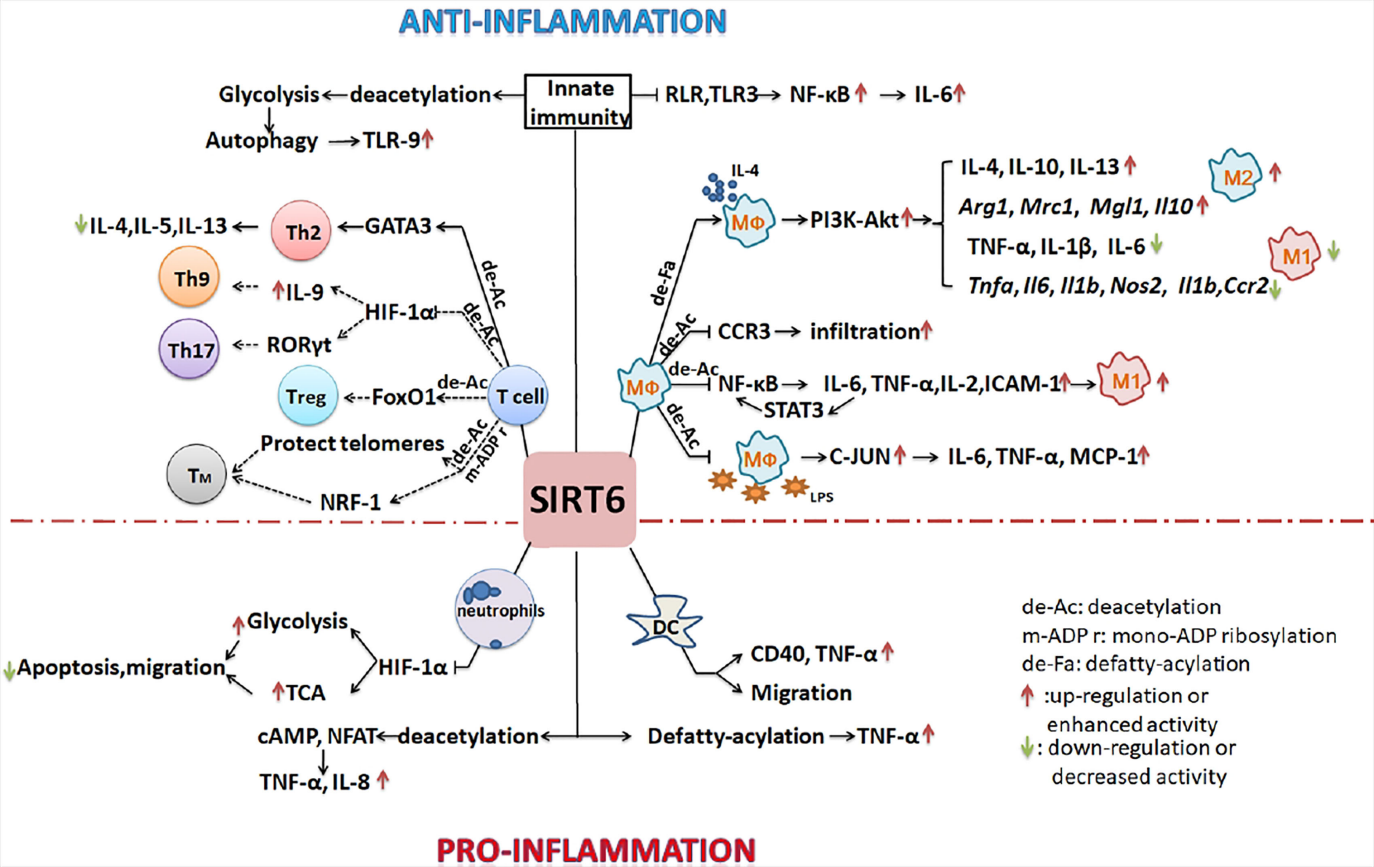
SIRT6 regulates the development of inflammation by regulating of the differentiation and function of immune cells. SIRT6 mainly inhibits the polarization of M1 macrophages, promotes the polarization of M2 macrophages, inhibits the overreaction of T cells, and suppresses innate immunity to inhibit the inflammatory process. By promoting the migration of neutrophils and DCs, it may also enhance inflammation. (Solid lines indicate that there is data support; dotted lines indicate a possible situation).

SIRT6 is a longevity protein that prevents cells, tissues, organs, and the body from aging. Although the mechanisms underlying these effects are diverse, they all involve resistance of aging by promoting of DNA damage repair, maintaining of the normal telomere structure of chromosomes, regulating of glucose and NAD^+^ metabolic balance, and by regulating of SASP (**Figure 1**). SIRT6 can also affect the differentiation and function of immune cells by regulating PTM affecting cells or the immunometabolism. However, the role of SIRT6 in immune regulation is complex. Although most studies have shown it to have anti-inflammatory activity, there is no lack of evidence regarding its pro-inflammatory potential (**Figure 2**). There has been insufficient research on how SIRT6 affects inflammation by regulating immune cells; SIRT6 has rarely been studied in many immune cells including granulocytes, monocytes, B cells, natural killer (NK) cells, and NKT cells. However, according to the recent research, the SIRT6-PTM or immunometabolism axes represent new directions with research potential. Further studies are required to clarify the role of SIRT6 in the regulation of inflammation, for example, its impact on different immune cells in different diseases or at different stages of aging, as well as on the differentiation, maturation, and function of immune cells.

The role of SIRT6 in cancer development is complex. SIRT6 shows differential expression in cancer tissues compared with normal tissues; its expression levels may also vary among different cancers, at different stages of the same cancer, and in different cell lines of the same tumor type. It also has both positive and negative effects on the regulation of cancer. Possible reasons for this include the following. 1. SIRT6 participates in the NAD^+^ decomposition pathway and regulates the activity of many proteins related to cancer developmental pathways by controlling levels of NAD^+^. Cancers that occur at different ages or in different stages of cancer development show different intracellular NAD^+^ concentrations; thus, the role of SIRT6 will be different. 2. As SIRT1 and SIRT7 are also located in the nucleus, they may compete with SIRT6 for NAD^+^ consumption and also, importantly, regulate PTMs, thereby affecting cancer-related signaling pathways. The dynamic changes in SIRT1, SIRT6, and SIRT7 levels in different cancers, as well as their PTMs on histones and non-histone substrates, increase the complexity of the role of SIRT6 in pathological processes. To elucidate the complex influence of SIRT6 on cancer, it will be necessary to carry out more studies focusing on specific patient ages and tumor stages.

Few studies have analyzed whether SIRT6 could achieve anti-cancer effects *via* regulation of immune cell function. This could represent a new direction for future research. For example, it may be possible to adjust the polarization of macrophages through SIRT6 to affect tumor progression. In the healthy state, higher SIRT6 levels promote the polarization of M2 macrophages and maintain a low level of inflammation, which can prevent chronic inflammation and cancer development. On the other hand, in the initial stage of cancer, reduced SIRT6 levels promote the polarization of M1 macrophages. This in turn increases the pro-inflammatory ability of macrophages, leading to more immune cells being recruited to the cancer tissue to eliminate cancer cells by forming a strong immune protective barrier. In the stage of primary tumor formation and the development of aggressive tumors, reducing SIRT6 levels can increase the M1/M2 ratio, preventing the formation of an immunosuppressive tumor microenvironment and thereby inhibiting tumor development and invasion. Such tumor immunotherapy needs to be adjusted according to the progression of cancer and the different types of immune cells. More research is needed to further understand the regulatory role of SIRT6 in the immune system and in cancer.

As SIRT6 can regulate immune cell function, it could also promote or inhibit cancer development by influencing cancer cell metabolism, survival, proliferation, apoptosis, migration, and other pathways. Therefore, when designing SIRT6 activators or inhibitors to treat cancer, comprehensive consideration is necessary of the differential impact on cancer cells and immune cells to avoid conflicting drug effects. Precise administration using cell-targeted drugs is a potential approach.

Taken together, these findings indicate that SIRT6 will serve as an important target candidate for regulating immunosenescence and immune cell function. Drugs designed to target SIRT6 will also make an important contribution to the fight against chronic inflammation and cancer. SIRT6, as an important regulator throughout immunosenescence, inflamm-aging, and cancer, is a potential target for the regulation of the immune system.

## Author Contributions

YW, JJ, and YL figured out the idea of writing this review. YL summarized the published results and drafted the manuscript. YW and JJ revised the manuscript. All authors contributed to the article and approved the submitted version.

## Funding

This project was supported in partial by the National Natural Science Foundation of China, No. 21977121 (YW) and in partial by important Direction Project Cultivation Fund, Institute of Immunology and the CAS Key Laboratory of Innate Immunity and Chronic Disease (2020) & University of Science and Technology of China (2021) (YW).

## Conflict of Interest

The authors declare that the research was conducted in the absence of any commercial or financial relationships that could be construed as a potential conflict of interest.

## Publisher’s Note

All claims expressed in this article are solely those of the authors and do not necessarily represent those of their affiliated organizations, or those of the publisher, the editors and the reviewers. Any product that may be evaluated in this article, or claim that may be made by its manufacturer, is not guaranteed or endorsed by the publisher.
